# Population kinetics during simultaneous infection of insect cells with two different recombinant baculoviruses for the production of rotavirus-like particles

**DOI:** 10.1186/1472-6750-7-39

**Published:** 2007-07-04

**Authors:** Jimmy A Mena, Octavio T Ramírez, Laura A Palomares

**Affiliations:** 1 Departamento de Medicina Molecular y Bioprocesos, Instituto de Biotecnología, Universidad Nacional Autónoma de México (UNAM), Apdo. Postal. 510-3. Cuernavaca, Morelos, CP. 62250, México

## Abstract

**Background:**

The simultaneous production of various recombinant proteins in every cell of a culture is often needed for the production of virus-like particles (VLP) or vectors for gene therapy. A common approach for such a purpose is the coinfection of insect cell cultures with different recombinant baculoviruses, each containing one or more recombinant genes. However, scarce information exists regarding kinetics during multiple infections, and to our knowledge, no studies are available on the behavior of the different populations that arise during coinfections. Such information is useful for designing infection strategies that maximize VLP or vector yield. In this work, kinetics of cell populations expressing rotavirus GFPVP2 (infected with bacGFPVP2), VP6 (infected with bacVP6), or both proteins simultaneously (coinfected with both baculoviruses) were followed by flow cytometry.

**Results:**

In single infections, the population infected with any of the recombinant baculoviruses followed the Poisson distribution, as the population expressing a recombinant protein exhibited a hyperbolic-type function with respect to the multiplicity of infection (MOI) up to 5 pfu/cell. In coinfections, the population fraction expressing each recombinant protein could not be anticipated from results of single infections, as in some cases interference and synergistic effects were found. Only cultures with a total MOI below 5 pfu/cell followed the Poisson distribution. For cultures with a MOI of bacGFPVP2 above that of bacVP6 (overall MOI above 5 pfu/cell), the total population expressing one or both recombinant proteins was as low as 50% below that predicted by Poisson. In contrast, the population fraction expressing VP6 increased in coinfections, compared to that in single infections. The largest population fraction simultaneously expressing both recombinant proteins was 58%, and corresponded to cultures infected at a MOI of 5 and 1 pfu/cell of bacGFPVP2 and bacVP6, respectively.

**Conclusion:**

The infection conditions that maximize the cell population simultaneously expressing two recombinant proteins were determined. Such conditions could not have been anticipated from population kinetics in individual infections. This information should be taken into account for improved simultaneous production of various recombinant proteins in any work dealing with coinfections.

## Background

Virus-like particles (VLP) are structurally identical to native viruses, but they lack the viral genetic material [1]. VLP are obtained when the major viral structural proteins are simultaneously expressed in a recombinant system. There exists an increasing interest on VLP production due to their promising applications as vaccines, as delivery vehicles for substances or genes, or as biosensors [1]. A recent example of the importance of VLP is the recent approval of Merck's vaccine against human papilloma virus. The production of VLP is a complex process and a challenging task, as it requires the simultaneous expression of various recombinant proteins. Due to its versatility and simplicity for coexpressing various recombinant genes, the insect-cell baculovirus expression vector system (IC-BEVS) has been commonly employed for producing VLP of several viruses.

The simultaneous production of several proteins in insect cells requires the delivery of various genes, either by a number of individual baculoviruses or by employing a single virus that contains several genes [2-4]. Of these strategies, the use of individual baculoviruses allows the manipulation of the concentration of each protein by changing the multiplicity of infection (MOI) of each virus [3,5,6]. In this way, the stoichiometry between the structural proteins may be controlled. In some VLP, which can have variable protein composition, changes in the ratio between structural proteins result in different VLP compositions, which can yield particles with different immunogenicity [5,6]. It is also possible that different stoichiometries between the structural proteins result in changes in VLP assembly efficiencies or kinetics, although this remains to be studied. Therefore, MOI manipulation is a powerful tool for finding the conditions required for maximizing the assembly of a desired VLP. However, little is known about the performance of simultaneous infections with various recombinant baculoviruses, specifically regarding cell population kinetics and possible interferences or synergies between the coinfecting viruses.

Rotavirus is a triple-layered virus that is responsible of gastroenteritis. The inner layer, a core-like particle, is constituted by VP2, surrounded by a second concentric layer containing VP6. The third layer is formed by VP7 and spikes of VP4 [7]. Recently, Mena et al. [8] studied the accumulation in insect cells of double layered rotavirus-like particles (dlRLP), that are constituted by the two inner concentric layers. They found that the assembly of dlRLP occurs intracellularly, and that, when expressed individually, both VP2 and VP6 form structures that cannot further assemble into double-layered particles. Namely, under such condition VP6 forms tubes whereas VP2 forms core-like particles densely packed in ordered groups. Neither tubes nor densely packed cores are accessible for interaction with the other recombinant protein, and thus dlRLP cannot be formed. These findings underline the importance of having both VP2 and VP6 available for assembly into dlRLP in each cell of the culture and at the right time to avoid the formation of structures composed only by a single protein. A similar situation occurs when other VLP are produced, such as adeno-associated viral vectors, which require the simultaneous expression of the structural and non-structural proteins, as well as replication of vector DNA in the same cell [9]. It can be anticipated that appropriate infection strategies, based on the manipulation of MOI, would maximize VLP yield. Several groups have studied the dynamics of infection at various MOI, while others have predicted by mathematical modeling the percentage of the population infected under different conditions [2-5,10-14]. Nonetheless, cell population kinetics during the simultaneous infection with two recombinant viruses have, to our knowledge, not been studied

A powerful tool for assessing the dynamics of infection at different MOI is flow cytometry. This methodology has been used to determine the percentage of the population infected at different MOI by a single type of virus [15,16], for assessing the productivity of different cell lines [17], to measure respiratory activity [18], to titer viruses [19], and to determine transducing titers of gene delivery vectors [20]. Flow cytometry has also been used to screen BF2 fish cells simultaneously infected with non-recombinant viruses of two different species; the pancreatic necrosis virus and the hematopoietic necrosis virus [21]. In our work, insect cells were infected with one or two different recombinant baculoviruses, expressing rotavirus VP2 or VP6, at different MOI. Utilizing flow cytometry we have, for the first time, determined the kinetics of cell populations expressing either of the recombinant proteins during simultaneous infections with both recombinant baculoviruses and compared them with single infections. Moreover, we have determined the infection conditions required to guarantee that the highest fraction of the population is simultaneously expressing both recombinant proteins, one of the necessary conditions for complete and efficient dlRLP production.

## Results

### Cultures infected with a single baculovirus

**Figure 1:**
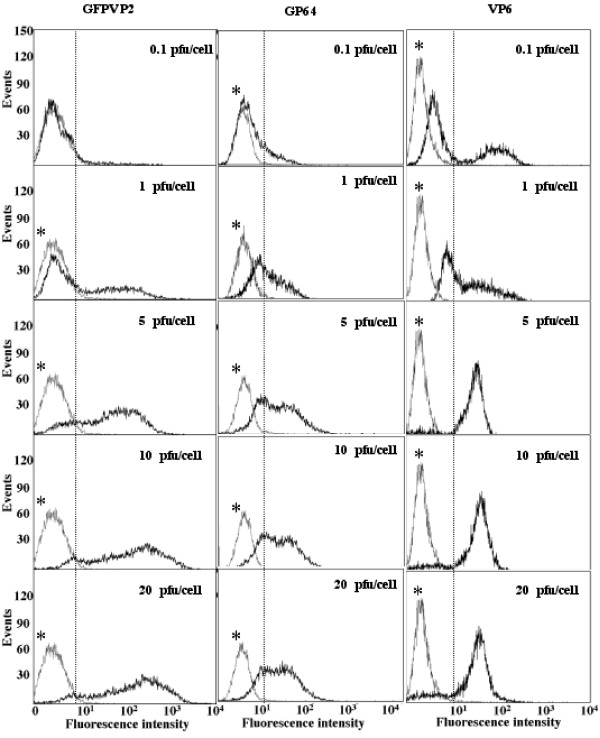
**Fluorescence histogram analysis of cells infected with bacGFPVP2 or bacVP6 at different multiplicities of infection at 24 hpi**. Fluorescence of GFPVP2, immunolabeled gp64 in cells expressing GFPVP2, and immunolabeled VP6 are shown in black. A control-uninfected culture is shown in gray and marked as (*). Dotted vertical line corresponds to the fluorescence gating used.

**Figure 2:**
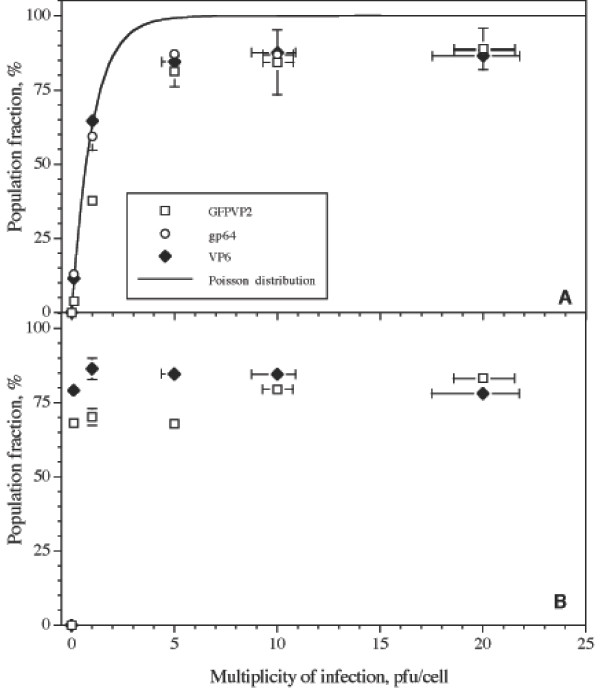
**Cultures individually infected with bacGFPVP2 or bacVP6**. Both sets of cultures are plotted in the same graphs to allow comparison. A. Fraction of the population expressing GFPVP2, gp64, or VP6 at 24 hours post infection (hpi). The probability (*p*) of a population of being infected by *w *virus, predicted by Poisson, is shown as a solid line. B. The same cultures as in A but at 48 hpi. Media and difference between duplicate cultures are shown in Y axis error bars. X error bars represent the standard deviation of titers of the viral stocks, adjusted to each MOI. Some error bars are smaller than the symbols shown.

Insect cell cultures individually infected with bacGFPVP2 or VP6 were analyzed by flow cytometry at 24 and 48 hours post-infection (hpi). MOI of 0.1, 1, 5, 10, and 20 pfu/cell were tested. Fluorescence histograms at 24 hpi are shown in Figure 1, along with an uninfected control culture performed simultaneously. When analyzing these results, it should be taken into account that fluorescence emission was measured at two different wavelengths, 510 for GFPVP2 and 575 nm for immunolabeled gp64 and VP6. Thus, background fluorescence of uninfected cells was different. Cells expressing a recombinant protein could be easily distinguished from uninfected cells. The distribution of the populations expressing GFPVP2 was different from the one expressing VP6. Data extracted from fluorescence histograms of duplicate cultures are summarized in Figure 2A, where the fraction of the population expressing a recombinant protein is reported relative to the total cell count (regardless of viability). It should be considered that the analysis shown in Figure 1 allows the identification of the population expressing a recombinant protein (either GFPVP2 or VP6), but not necessarily that of infected cells, as expression of the recombinant gene might be absent or inefficient even in infected cells. A marker that has been used to monitor infection by baculovirus is gp64 [22]. gp64 is the major envelope glycoprotein of baculovirus [23], and accumulates in the membrane of infected cells during the first 10 to 12 hpi [24]. To correlate infection with recombinant protein expression, gp64 was immunodetected at 24 hpi in cultures expressing GFPVP2 (Figures 1 and 2A). In addition, the population distribution predicted by Poisson:

p(w)=[(MOIww!)e-MOI]
 MathType@MTEF@5@5@+=feaafiart1ev1aaatCvAUfKttLearuWrP9MDH5MBPbIqV92AaeXatLxBI9gBaebbnrfifHhDYfgasaacH8akY=wiFfYdH8Gipec8Eeeu0xXdbba9frFj0=OqFfea0dXdd9vqai=hGuQ8kuc9pgc9s8qqaq=dirpe0xb9q8qiLsFr0=vr0=vr0dc8meaabaqaciaacaGaaeqabaqabeGadaaakeaacqqGWbaCdaqadaqaaiabbEha3bGaayjkaiaawMcaaGGaaiab=1da9maadmaabaWaaeWaaeaadaWcaaqaaiabb2eanjabb+eapjabbMeajnaaCaaaleqabaGaee4DaChaaaGcbaGaee4DaCNaeeyiaecaaaGaayjkaiaawMcaaiabbwgaLnaaCaaaleqabaGaeeyla0Iaeeyta0Kaee4ta8KaeeysaKeaaaGccaGLBbGaayzxaaaaaa@42B6@

commonly used to describe infection, is also plotted in Figure 2A[3]. The population fraction expressing any of the recombinant proteins or gp64 increased in a hyperbolic-type function with MOI up to 5 pfu/cell, and then remained constant for MOI above 5 pfu/cell. Such a behavior is in agreement with predictions based on the Poisson distribution. Cultures infected with bacVP6 or bacGFPVP2 had a similar trend, which was also similar to the population expressing gp64 in the cultures infected with bacGFPVP2. The similar behavior between cells expressing GFPVP2, gp64 and VP6 confirms that the results obtained from cells immunostained for VP6 were representative of the population expressing that recombinant protein, and that both recombinant baculoviruses were equally infective. Results obtained at 48 hpi are shown in Figure 2B. The Poisson distribution is not plotted in Figure 2B, as secondary infection had occurred at this time, and different calculations are needed to predict the infected population under these conditions (see discussion below). At 48hpi, between 65 to 90% of the cells were expressing either recombinant protein, regardless of the MOI. However, at MOI below 10 pfu/cell, a smaller population of cells expressed GFPVP2, in comparison with that expressing VP6.

**Figure 3:**
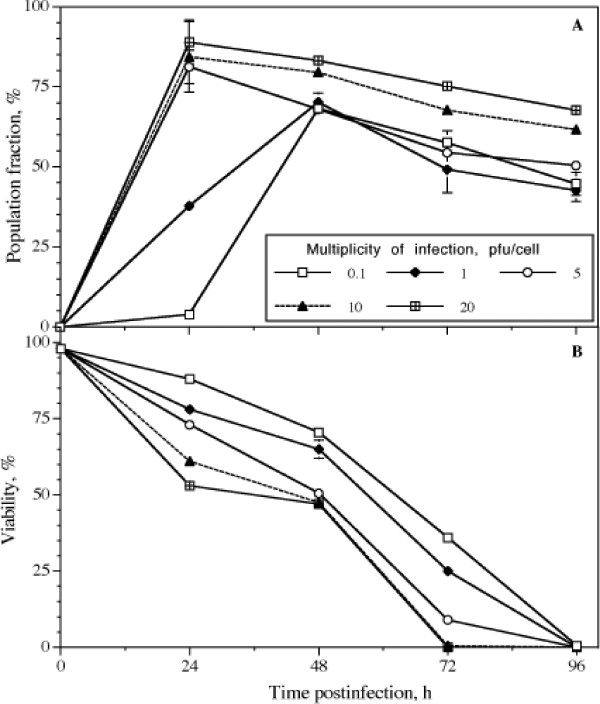
**Cultures infected only with bacGFPVP2**. A. Kinetics of the fraction of the population expressing GFPVP2 in cultures infected only with bacGFPVP2. Numbers in figure legend refer to the different MOI used. B. Viability of cultures infected with bacGFPVP2. Media and difference between duplicate cultures are shown. Some error bars are smaller than the symbols shown.

Handling of cultures expressing GFPVP2 for FACS analysis was easier than those for VP6, as VP6 had to be immunostained by a laborious procedure, whereas intrinsic fluorescence of GFPVP2 facilitated the assay. Therefore the population containing this recombinant protein was followed every 24 h until 96 hpi. Results are shown in Figure 3A. The population expressing GFPVP2 reached a maximum at 24 hpi for cultures infected at a MOI of 5 pfu/cell or higher. In contrast, such population reached a maximum until 48 hpi for cultures infected at MOI of less than 5 pfu/cell. In all cases, the percentage of the population expressing a recombinant protein decreased after 48 hpi (Figure 3B), probably due to degradation of the recombinant protein after loss of cell membrane integrity in non-viable cells. Viability decreased steadily after infection, reaching 0% at 72 hpi in cultures infected at MOI of 10 and 20 pfu/cell. The rate of decrease of the viability was lower as the MOI decreased.

### Cultures simultaneously infected with bacGFPVP2 and bacVP6

**Figure 4:**
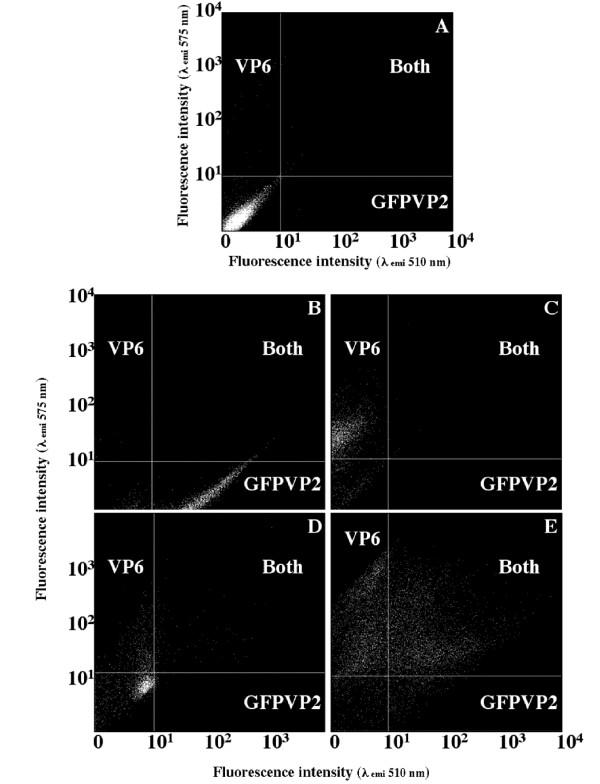
**Identification of cell populations simultaneously expressing GFPVP2 and VP6**. Cultures shown in panels A to C were used for setting the gating limits for analysis of coinfected cultures. A. Uninfected control culture. B. Culture at 24 hpi infected with bacGFPVP2 at a MOI of 5 pfu/cell. C. Culture at 24 hpi infected with bacVP6 at a MOI of 5 pfu/cell. D. Culture coinfected with bacGFPVP2 and bacVP6 with a MOI of 0.1 pfu/cell of each virus, 24 hpi. E. Culture coinfected with bacGFPVP2 and bacVP6 with a MOI of 0.1 pfu/cell of each virus, 48 hpi.

Gating parameters used for discriminating between cells expressing GFPVP2, VP6 or both proteins were set according to fluorescence (emission) at 510 nm (corresponding to GFP) and 575 nm (corresponding to R-phycoerythrin, used to detect VP6) in uninfected cultures, cultures expressing only GFPVP2, or cultures expressing VP6. Typical results are shown in Figures 4A–C. As expected, uninfected cultures had a low emission both at 510 or 575 nm (Figure 4A), whereas cells infected with bacGFPVP2 had a high emission at 510 nm (Figure 4B). Moreover, cells infected with bacVP6 had a high fluorescence at 575 nm (Figure 4C). Accordingly, gating parameters, which allowed the differentiation between the three populations present in coinfected cultures, could be set. Flow cytometry results of a culture simultaneously infected with bacGFPVP2 and VP6 at 24 or 48 hpi are shown in Figure 4D and 4E, respectively. At 24 hpi many cells could be clearly classified as expressing VP6, whereas only relatively few cells were expressing both recombinant proteins. At 48 hpi, only a few cells were not expressing a recombinant protein, while most were either expressing VP6 or both recombinant proteins simultaneously. A small fraction of the population was expressing only GFPVP2. A quantitative analysis of the flow cytometry study is presented below.

**Figure 5:**
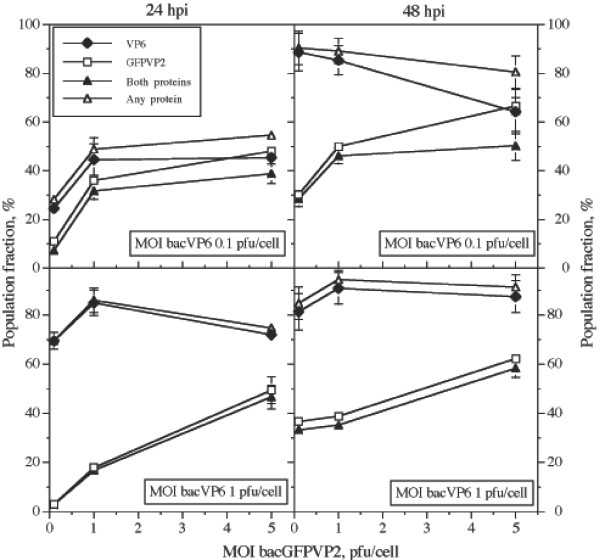
Population expressing GFPVP2, VP6, both recombinant proteins, or any recombinant protein (either GFPVP2 or VP6, or both). Media and difference between duplicate cultures are shown. Some error bars are smaller than the symbols shown.

The results obtained from 4 experimental setups performed in duplicate are summarized in Figure 5. Cultures were infected at different MOI combinations of bacGFPVP2 and bacVP6. MOI of each virus were not increased above 5 pfu/cell, as higher MOI did not result in a higher population fraction expressing any of the recombinant proteins in single infections (Figure 2). In most cases, the largest fraction of the population expressed VP6, regardless of the MOI of bacGFPVP2. The only exceptions were the cultures infected with 0.1 and 5 pfu/cell of bacVP6 and bacGFPVP2, respectively, either at 24 or 48 hpi. In these cultures, the population expressing either of the recombinant proteins was about the same. It should be noted that, in the extreme situation, the population fraction expressing VP6 was 17.5 times larger than that expressing GFPVP2 (MOI bacVP6 1 pfu/cell, MOI bacGFPVP2 0.1 pfu/cell, 24 hpi). The highest percentage of the population expressing any recombinant protein (VP6 or GFPVP2 or both) at 24 hpi was obtained at MOI of 1 pfu/cell for both bacGFPVP2 and bacVP6, i.e. a total MOI of 2 pfu/cell. Such population fraction was 15% higher than that expressing any recombinant protein at the highest total MOI tested, which corresponded to 6 pfu/cell (5 pfu/cell of bacGFPVP2 and 1 pfu/cell of VP6). The population fraction expressing both recombinant proteins at 24 hpi closely followed the population expressing GFPVP2.

Coinfections had various effects on the population expressing GFPVP2. In general, the cell population expressing GFPVP2 in coinfected cultures was smaller than in single-infected cultures at the same MOI, except in two cases at 24 hpi (Figures 2 and 5). Namely, in cultures infected at a MOI of 0.1 pfu/cell of each baculovirus, the population expressing GFPVP2 was duplicated, in comparison to that observed in single infections. Moreover, at 0.1 pfu/cell of bacVP6 and 1 pfu/cell of bacGFPVP2, the population expressing GFPVP2 was the same than that observed in single infections. In contrast, the population expressing VP6 at 24 hpi increased in coinfections, compared to individual infections. In cultures infected at MOI of 0.1 pfu/cell of bacVP6, 11.5% of the population expressed VP6 in single-infected cultures, whereas in coinfections up to 45% of the population expressed VP6 (Figures 2 and 5). Something similar occurred at a MOI of bacVP6 of 1pfu/cell, as 65% of the population expressed VP6 in single-infected cultures, while in coinfections such percentage increased to 70 – 85% at 24 hpi.

Compared to 24 hpi, the population expressing any of the recombinant protein increased at 48 hpi (Figure 5). This increase occurred even in cultures infected with a MOI of bacGFPVP2 of 5 pfu/cell; a behavior not observed in individual infections. The population fraction expressing VP6 at 48 hpi reached 98% (considering the error bars) in cultures infected at MOI of 0.1 or 1 pfu/cell of any of the baculoviruses. In most cases the percentage of the population expressing GFPVP2 at 48 hpi was smaller than that expressing VP6, with the exception of the cultures infected with 0.1 pfu/cell of bacVP6 and 5 pfu/cell of bacGFPVP2, where the population expressing either protein was equal. The condition that resulted in the highest percentage of the population simultaneously expressing both recombinant proteins was a MOI of bacGFPVP2 of 5 pfu/cell and 1 pfu/cell of bacVP6. Such condition resulted in 48% and 58% of the population at 24 and 48 hpi, respectively, simultaneously expressing both recombinant proteins. Populations expressing the recombinant proteins were not followed after 48 hpi as further infections by progeny viruses were not expected after 48 hpi, and a rapid decline in viability was observed after this time (data not shown).

**Figure 6:**
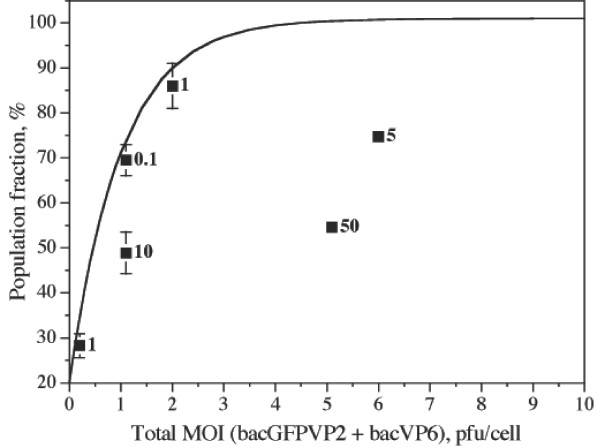
**Comparison of coinfected cultures at 24 hpi with the Poisson distribution**. The population fraction expressing any of the recombinant proteins (either GFPVP2 or VP6, or both) is shown. Numbers at the right of symbols are the MOI ratio between bacGFPVP2 and bacVP6.

To better appreciate the effect of coinfections, the population expressing any of the recombinant proteins (GFPVP2, VP6, or both) at 24 hpi is shown in Figure 6 as a function of total MOI (MOI bacGFPVP2 + MOI bacVP6). For comparison, the Poisson distribution is also plotted. It can be seen that coinfected cultures with a MOI of bacVP6 equal or higher than that of bacGFPVP2 followed the Poisson distribution. However, cultures where the MOI of bacGFPVP2 was 5, 10, or 50 times higher than that of bacVP6 had a much lower population expressing any of the recombinant proteins than that predicted by the Poisson distribution at the corresponding cumulative MOI.

## Discussion

Previous reports [15-17] and this work demonstrate the utility of flow cytometry for monitoring recombinant protein expression. In this work, a wider screening of recombinant protein expression at different MOI and at different times postinfection was performed, in addition to following the populations expressing two recombinant proteins during coinfections. At 24 hpi, the percentage of the population expressing VP6 and gp64 closely followed the Poisson distribution. This observation is relevant, as it has been shown that various culture variables, including medium composition, mode of culture, temperature, etc., can affect infection [25-27]. The percentage of the population expressing a recombinant protein did not reach 100%, most probably due to the viability of cultures at the time of infection, which was between 96 and 98%. It should be noted that the population expressing GFPVP2 at 24 hpi with a MOI below 5 pfu/cell was in most cases lower than that expressing gp64 (detected in the culture infected with bacGFPVP2) or VP6. It is possible that the smaller population expressing GFPVP2 was a result of the slower production rate of this protein in comparison with VP6, as has been observed previously [3,4,14]. Thus, in some cells the amount of GFPVP2 at 24 hpi may be below the detection limit of the flow cytometer. It should be taken into account that, according to the life cycle of baculovirus, production of GFPVP2 would start around 20 hpi, as its gene is under the very late *polh *promoter [24]. In contrast, the population expressing gp64, which is expressed in the early and late phases of infection (from 0 to 20–24 hpi), behaved as VP6, which is also under the *polh *promoter but has a higher production rate than GFPVP2 [3]. Thus, the observed difference cannot be attributed to a difference in infectivity between the two recombinant viruses.

Measurements performed at 24 hpi reflect primary infection. At 48 hpi, the population of cells expressing a recombinant protein increased in cultures infected at MOI below 5 pfu/cell. Such an increase was a result of infection by the viral progeny produced during the late phase of the primary infection (12–24 hpi, [24]). At 48 hpi, secondary infection resulted in a similar population of cells expressing VP6 regardless of differences in the MOI (Figure 2). In contrast, the population expressing GFPVP2 at MOI below 10 pfu/cell did not catch up at 48 hpi with that at the higher MOI of 10 or 20 pfu/cell. Such a lower percentage of population expressing GFPVP2 at 48 hpi may be a result of the rapid decline in viability observed in these cultures (Figure 3B), which did not occur in cultures expressing VP6 (data not shown). Wu et al. [28] also observed that infection with baculoviruses containing different recombinant genes results in different cell death kinetics. The decrease in viability was faster as the MOI increased, similarly to what Wu et al. [28] observed in the range of 0.5 to 10 pfu/cell.

In general, population fractions obtained from individually infected cultures cannot be extrapolated to coinfections, a phenomenon that has not been previously described in coinfections with two different recombinant viruses of the same species. The increase of the population expressing VP6 in coinfected cultures may be a result of a cooperative action between both baculoviruses. Cultures infected at low MOI of bacVP6 would result in only a fraction of the population infected with this virus. Progeny virus would begin to bud from 12 hpi [11] and infect cells still susceptible to additional infection. It has been shown that virus binding to infected cells can occur up to 24 hpi [10]. Therefore, cells initially infected with bacGFPVP2 were still susceptible to additional infection by bacVP6, but, in contrast to single infections at low MOI, all the viral proteins and transcription factors of the very late phase of infection would be already present when the VP6 gene reached the cell nucleus. This may occur as early as 1 hour after progeny virus budding [10]. Accordingly, transcription of the VP6 gene could start, during secondary infection, as early as 13 h after the initial infection by bacGFPVP2. This would explain the higher population expressing VP6 in coinfections in comparison to individual infections. According to this hypothesis, the difference between the populations expressing VP6 in coinfections or in single infections roughly corresponded to the fraction of cells simultaneously expressing both recombinant proteins. A different situation was observed in the case of the population expressing GFPVP2. At MOI of 5 pfu/cell, the population expressing GFPVP2 decreased around 30% in coinfections compared to single infections. In general, coinfections at high MOI of GFPVP2 resulted in a lower population expressing a recombinant protein. It appears that secondary infection of bacVP6 interferes with the expression of GFPVP2 by a mechanism still unknown. A similar phenomenon was observed by Alonso et al. [21], in, to our knowledge, the only other work characterizing coinfections with flow cytometry, although using non-recombinant viruses. In such work, coinfections with hematopoietic necrosis virus and hepatic necrosis virus were screened in a fish cell line. The hepatic necrosis virus interfered with growth of the hematopoietic necrosis virus. However, care should be taken when comparing our work with that of Alonso et al. [21], as they worked with two different virus species. Following mRNA kinetics may help elucidate the interference mechanism of bacVP6 with the expression of GFPVP2. Vieira et al. [4] followed the kinetics of baculovirus replication and found that the number of copies of baculovirus DNA coding for VP2 was about half of that coding for VP6 in coinfected cultures, which could explain a less efficient secondary infection by bacGFPVP2. They also found that no difference exists between the mRNA stability of VP2 or VP6 expressed in the insect-cell baculovirus system. It remains to be determined if the same situation occurs in the case of the fusion gene GFPVP2.

We have previously compared the production of VP2 and VP6 upon infection or coinfection of SF9 cells [3]. We found that the production rates of VP2 and VP6 were similar in individual infections or coinfections at an MOI of 5 pfu/cell. In this work, we did not follow the recombinant protein production rates. However, we did observe that in the culture coinfected with 5 pfu/cell of bacGFPVP2 and 1 pfu/cell of bacVP6 (the closest conditions to our previous study), the population fraction expressing each of the recombinant proteins was similar to that observed in individually infected cultures. It can be inferred that such cultures would also have a similar recombinant protein production rate, as the cultures we have coinfected in the past at a MOI of 5 pfu.cell.

For efficient production of rotavirus like-particles, adequate VLP assembly would require the simultaneous expression of both GFPVP2 and VP6 by the largest population of cells [8]. Such situation was encountered at a MOI of 5 pfu/cell of bacGFPVP2 and 1 pfu/cell of bacVP6, with 58% of the population expressing both recombinant proteins at 48 hpi. It was observed that the population simultaneously expressing both recombinant proteins closely followed the population expressing GFPVP2, indicating that expression of bacGFPVP2 was the limiting step. We are now working on kinetic studies of the production of single- or double shelled rotavirus-like particles upon infection with bacGFPVP2 and bacVP6.

The characteristics of a recombinant protein and expression system directly affect the production kinetics and determine the limiting steps during its biosynthesis. For instance, molecular weight, posttranslational modifications, the site of accumulation, and other characteristics can affect protein yields. Accordingly, the results presented here can serve as a general practical guideline but inherent characteristics of each particular case must be considered.

## Conclusion

In this work we have, for the first time determined the effect of the simultaneous infection with two recombinant baculovirus on the populations expressing two recombinant proteins. The kinetics of cell populations expressing both recombinant proteins in coinfections could not have been predicted from single infection data or from the Poisson distribution based on theoretical considerations. It was observed that secondary infection of bacVP6 interfered with bacGFP2 expression, and also resulted in a higher percentage of the population expressing VP6. The MOI for each virus that resulted in the highest population percentage expressing both recombinant proteins was found. The information generated in this work describes a novel phenomenon and is useful for designing rational infection strategies needed in several applications for improving the simultaneous expression of two recombinant proteins in systems based on viral gene delivery. Future work in coinfections with several recombinant baculoviruses for producing various proteins by insect cells should take into account that the population behavior of individual infections may be different to that in single infections.

## Methods

### Cell culture and recombinant protein expression

High Five cells (Invitrogen, Carlsbad, CA, USA) were cultivated in suspension in SF900-II medium (Invitrogen, Carlsbad, CA, USA) in 250 mL shaker flasks with 60 mL of working volume at 27°C and 115 rpm. Cell concentration and viability prior to infection were determined with a Coulter Counter (Coulter Instruments) and Trypan blue exclusion in a hemacytometer, respectively. Two recombinant baculoviruses derived from *Autographa californica *nucleopolyhedrovirus (*Ac*MNPV) were used. One baculovirus contained the gene for the fusion protein GFPVP2 (bacGFPVP2, kindly provided by Prof. J. Cohen, INRA, France [29]), and the other contained the gene of VP6 (bacVP6, strain SA11, kindly provided by Dr. S. López, IBT-UNAM, Mexico). Both recombinant genes were under the *polh *promoter. Viral stocks were titrated as described in Mena et al. [30]. This method has typical standard deviations between 10 and 30%. Cultures were infected at a cell concentration of 0.5 × 10^6 ^cell mL^-1 ^using the different MOI described in the Result section.

### Cell preparation for flow cytometry analysis

To obtain the required cell number for flow cytometry analysis, 15 mL of culture infected with either bacVP6, bacGFPVP2 or co-infected were centrifuged at 1,000× g for 10 min in an Eppendorf 5810R centrifuge (Hamburg, Germany) at 4 °C degrees. The supernatant was discarded and the pellet was washed with 15 mL of PBS and centrifuged at 1,000× g for 10 min. The pellet was then fixed with 15 mL of 2% formaldehyde (Sigma-Aldrich, St, Louis, MO, USA) in PBS for 15 min, and washed twice with PBS. Cells were permeabilized for 15 min with 0.2% sodium deoxycholate (Sigma-Aldrich, St. Louis, MO, USA) in PBS with 2% bovine serum albumin (Sigma-Aldrich, St, Louis, MO, USA), and washed twice with PBS. For immunodetection of gp64 and VP6, one mL of pellet was resuspended in 1 mL of PBS (0.2% BSA) with primary antibody in dilution of 1:200 for 1 hr. The primary antibodies used were a monoclonal antibody to VP6 (Clone 3C10, Biodesign, Saco, MA, USA), and a monoclonal antibody Fastplax™ to gp64 (Novagen, Darmstadt, Germany). The cells were washed twice and resuspended in 1 mL of PBS (0.2% BSA) with the secondary antibody at a dilution of 1:200 for 1 h. The secondary antibody was a goat anti-mouse coupled to R-phycoerythrin (Molecular Probes-Invitrogen, Carlsbad, CA, USA). The cells were washed twice with PBS and resuspended in 500 μL of PBS.

### Flow cytometry analysis

A FACSort with CellQuest software (Becton-Dickinson, Franklin Lakes, NJ, USA) was used. The FACSsort has one light source (488 nm) with three-color fluorescence analysis. 100 μL of cell preparation was diluted with 400 μL of PBS and analyzed in the FACS. Cells expressing GFPVP2 were identified by GFP fluorescence (λ excitation: 488 nm, λ emission: 510, FL1 channel). Cells expressing gp64 or VP6 were analyzed by R-phycoerythrin fluorescence (λ excitation: 488 nm, λ emission: 575, FL2 channel). In all cases, 10,000 events were analyzed.

## Authors' contributions

JAM performed all the experiments, participated in experiment design and in data analysis and interpretation, and revised the manuscript critically.

OTR participated in experiment design, data analysis and interpretation, critically revised the manuscript, and provided important intellectual content.

LAP participated in the conceptual design of the experiments, in data analysis and interpretation, and drafted the manuscript.

All authors read and approved the final manuscript.
